# A Comparison of Face-Down Positioning and Adjustable Positioning After Pars Plana Vitrectomy for Macular Hole Retinal Detachment in High Myopia

**DOI:** 10.3389/fmed.2022.780475

**Published:** 2022-02-16

**Authors:** Yan Gao, Ting Ruan, Nan Chen, Bin Yu, Xiaoli Xing, Qing Du, Yan Qi, Jun Li

**Affiliations:** ^1^Qingdao Eye Hospital of Shandong First Medical University, Qingdao, China; ^2^State Key Laboratory Cultivation Base, Shandong Provincial Key Laboratory of Ophthalmology, Shandong Eye Institute, Shandong First Medical University & Shandong Academy of Medical Sciences, Qingdao, China

**Keywords:** adjustable positioning, face-down positioning, high myopia, macular hole, retinal detachment

## Abstract

**Purpose:**

To compare the anatomical and functional outcomes of macular hole retinal detachment (MHRD) in high myopia after pars plana vitrectomy (PPV) with face-down positioning and adjustable positioning.

**Methods:**

Fifty-three eyes from 53 patients with MHRD were analyzed in this study. All patients received PPV with silicon oil for tamponade and then subdivided into 2 groups: 28 were included in a face-down positioning group and 25 were included in the adjustable positioning group. Patients were followed up for at least 6 months. The main outcome was the rate of anatomical macular hole (MH) closure and retinal reattachment. Secondary outcome measures were the best-corrected visual acuity and postoperative complications.

**Results:**

There was no significant difference in the rate of MH closure (53.6 vs. 72.0%, *p* = 0.167) and retinal reattachment (100 vs. 96%, *p* = 0.472) between the face-down group and adjustable group. Compared with the mean preoperative best-corrected visual acuity (BCVA), the mean postoperative BCVA at the 6-month follow-up improved significantly in both groups (*p* = 0, both). But there was no significant difference in the mean postoperative BCVA (*p* = 0.102) and mean BCVA improvement (*p* = 0.554) at 6 months after surgery between the two groups. There was no significant difference in the high intraocular pressure (IOP) after surgery between the two groups (53.6 vs. 44%, *p* = 0.487). There were no other complications that occurred during the follow-up.

**Conclusion:**

Adjustable positioning after PPV with silicon oil tamponade for MHRD repair is effective and safe. Face-down positioning does not seem to be necessary for all patients with MHRD.

## Introduction

Macular hole retinal detachment (MHRD) is a serious vision impairment complication associated with high myopia. The pathogenesis of MHRD is not completely clear; however, it is believed that the tangential macular traction by the vitreoretinal interface, remnants of the cortical vitreous, inflexible internal limiting membrane (ILM), and the retinal vasculature is one of the factors (1, 2). In addition, the weakened retinal adherence to the posterior pole caused by choroidal and retinal pigment epithelium (RPE) atrophy is also one of the factors (3). Since it was first described by Gonvers and Machemer, pars plana vitrectomy (PPV) procedures have been used in the surgical treatment of MHRD with high myopia (4). Vitrectomy combined posterior vitreous cortex removal, epiretinal membrane removal, and ILM removal, with gas or silicone oil tamponade to become the standard treatment for MHRD with a higher retinal reattachment rate (5). Since Michalewska et al. first presented the inverted ILM flap technique (6), modified techniques, such as temporal ILM flap or inverted ILM insertion, have been introduced to potentially improve the surgical outcomes in MH and MHRD (7–20), or to enhance the success rate in eyes with persistent full-thickness macular hole undergoing secondary PPV (21).

More than 90% of vitreoretinal surgeons worldwide recommend some period of face-down positioning after macular hole (MH) repair surgery (22). However, it is a tough challenge for most patients to keep a strict face-down positioning after operation for a long time. Elderly patients or patients with systemic diseases have serious difficulties persisting in the face-down positioning. Furthermore, some rare postoperative complications, like ulnar nerve palsies, pulmonary embolism, thrombophlebitis, and decubitus, would develop after a long period of face-down position (23, 24). Multiple groups have reported the efficacy of postoperative positioning without the maintenance of a face-down positioning after vitrectomy for MH (22, 25–30) and retinal detachment (RD) (31–35). However, MHRD is excluded from their observation. The purpose of the current study was to evaluate the 6-month outcomes of adjustable positioning compared to face-down positioning after PPV for MHRD in high myopia.

## Materials and Methods

This retrospective study analyzed a consecutive series of 53 eyes (53 patients) with MHRD in high myopia who underwent primary PPV between January 2018 and December 2019 at Qingdao Eye Hospital. The study followed the tenets of the Declaration of Helsinki and was approved by the Institutional Review Board of Qingdao Eye Hospital of Shandong First Medical University.

The inclusion criteria were as follows: (1) eyes with an axial length (AL) ≥26 mm; (2) the diagnosis of MRHD confirmed by optical coherence tomography (OCT) before surgery, and RD extending by more than 1 disk diameter around the full-thickness MH; and (3) the follow-up time is more than 6 months. Those eyes with previous vitreoretinal surgery, ocular trauma, and presence of peripheral retinal breaks before surgery, diabetic retinopathy, and other proliferative vitreoretinopathy were excluded.

The following general information was obtained for analysis: sex, age, systemic diseases, and bilaterality. All the patients accepted the preoperative and postoperative examinations that included best-corrected visual acuity (BCVA), intraocular pressure (IOP), slit-lamp examination, AL, B-ultrasound, fundus photography, and OCT. The decimal BCVA was converted to the logarithm of the minimum angle of resolution (logMAR) units for statistical analyses. The AL was measured using a Master 500 (Carl Zeiss, Germany). The area of the RD was determined by the images from a panoramic scanning laser ophthalmoscope (SLO) (Optos, Scotland), which was used to classify patients into those whose RD was within or beyond the vascular arcade. The presence of an MH, MH closure, and retina reattachment were evaluated in the OCT images (Optovue, USA).

### Surgical Technique

Pars plana vitrectomy (PPV) procedures were performed using a standard 25-gauge 3-port system (Constellation, Alcon, USA). Core vitrectomy was performed by intravitreal injection of triamcinolone acetonide to visualize the vitreous gel and the posterior hyaloid. Peripheral vitreous base vitrectomy was performed under scleral depression. After being stained with indocyanine green (ICG) for 30 s, the ILM was peeled over the entire macular area and inserted into the MH to fill the hole. Fluid–gas exchange with drainage of subretinal fluid through the MH was performed. Finally, silicone oil was filled in all patients.

Patients were subdivided into two groups according to the postoperative positioning based on the recommendation of the surgeon. Face-down, as a routine treatment, meant keeping a face-down positioning for at least 12 h per day for at least 1 month after the surgery. Patients were encouraged to stay face-down during sleeping hours, as long as possible. In the adjustable group, patients were in a non-recumbent positioning during the daytime and fall asleep in the lateral positioning at night.

The primary endpoint was the MH closure and anatomical reattachment rate at 6 months after surgery. Secondary endpoints included BCVA change, change of IOP, and frequency of reported complications.

### Statistical Analysis

Data were analyzed using the chi-square test and the Fisher exact test for categorical variables, and the *t*-test and the Mann–Whitney U test for numerical and ordinal variables. A *P*-value of 0.05 was considered statistically significant.

## Results

A total of 53 eyes of 53 patients (28 eyes in the face-down group and 25 eyes in the adjustable group) were analyzed. They were 49 women and 4 men with a mean (±SD) age of 62.4 ± 8 years (range 40–81 years). There are more female patients (96.4% in the face-down group vs. 88% in the adjustable group, *p* = 0.523) and more right eyes (78.6 vs. 60%, *p* = 0.142) in both groups. The mean preoperative axial length was 29.98 ± 2.01 mm with a range of 26.54 to 35.82 mm. No significant differences in baseline parameters were found between the two groups (*p* > 0.05), including age, gender, systemic diseases (including hypertension, diabetes, coronary heart disease, and asthma), bilaterality, axial length, preoperative BCVA, and IOP. There were also no significant differences in the status related to retinal detachment between the two groups (*p* > 0.05), including whether it is an extent of RD beyond vascular arcade (64.3 vs. 68%, *p* = 0.776), combined with choroidal detachment (10.7 vs. 12%, *p* = 1), combined with macular membrane (32.1 vs. 20%, *p* = 0.317), and combined with lattice degeneration (46.4 vs. 44.0%, *p* = 0.859). There was no significant difference in the number of lens surgery and silicone oil volume between the groups (*p* > 0.05). The baseline parameters, status, and surgical procedure of the patients for the two groups are listed in Table 1.

**Table 1:** The baseline parameters, status, and surgical procedure of patients.

	**Total**	**Face-down** **group**	**Adjustable** **group**	**P value**
No. of eyes/patients	53	28	25	
Age (year) (mean ± SD; range)	62.4 ± 8.0 (40–81)	63.7 ± 5.8 (52–75)	61.0 ± 9.9 (40–81)	0.237
**Gender, no. (%)**				
Men	4 (7.5)	1 (3.6)	3 (12.0)	0.523
Woman	49 (92.5)	27 (96.4)	22 (88.0)	
Systemic disease, no. (yes/no)	30/23	18/10	12/13	0.232
**Eyes, no. (%)**				
Right	37 (69.8)	22 (78.6)	15 (60.0)	0.142
Left	16 (30.2)	6 (21.4)	10 (40.0)	
Axial length (mm) (mean ± SD; range)	29.98 ± 2.01 (26.54–35.82)	29.99 ± 1.76 (27.50–33.30)	29.96 ± 2.28 (26.54–35.82)	0.970
**Preoperative BCVA**				
LogMAR (mean ± SD; range)	2.25 ± 0.76 (0.7–4.0)	2.27 ± 0.71 (1.3–3.0)	2.22 ± 0.84 (0.7–4.0)	0.692
Preoperative IOP (mmHg) (mean ± SD; range)	12.7 ± 3.1 (6–19)	12.1 ± 2.7 (7–17)	13.4 ± 3.4 (6–19)	0.137
**Lens status, no. (%)**				
Phakia	45 (84.9)	26 (92.9)	19 (76.0)	0.147
Pseudophakia	6 (11.3)	1 (3.6)	5 (20.0)	
Aphakia	2 (3.8)	1 (3.6)	1 (4.0)	
Beyond vascular arcade, no. (%)	35 (66)	18 (64.3)	17 (68.0)	0.776
Combined choroidal detachment, no. (%)	6 (11.3)	3 (10.7)	3 (12.0)	1.000
Combined macular membrane, no. (%)	14 (26.4)	9 (32.1)	5 (20.0)	0.317
Combined lattice degeneration, no. (%)	24 (45.3)	13 (46.4)	11 (44.0)	0.859
**Lens surgery, no.(%)**				
No	31 (58.5)	16 (57.1)	15 (60.0)	0.179
Phaco	20 (37.7)	12 (42.9)	8 (32.0)	
Phaco+IOL implantation	2 (3.8)	0 (0.0)	2 (8.0)	
Silicone oil volume (ml) (mean ± SD; range)	6.7 ± 1.2 (4.3–9.5)	6.7 ± 1.2 (4.5–9.5)	6.7 ± 1.4 (4.3–9.0)	0.944

*BCVA, best corrected visual acuity; ILM, internal limiting membranes; IOL, intraocular lens; IOP, intraocular pressure; LogMAR, logarithm of the minimum angle of resolution*.

We collected the 6-month visual and anatomic outcomes for all the patients (as shown in Table 2). There was no significant difference in the mean postoperative BCVA at 6 months after surgery between the two groups (*p* = 0.102). Compared with the mean preoperative BCVA, the mean postoperative BCVA at the 6-month follow-up improved significantly in both groups (*p* = 0 in both groups), but no significant difference in mean BCVA improvement was found between the two groups (*p* = 0.554).

**Table 2:** Visual and anatomic results at 6 months after surgery.

	**Face-down group**	**Adjustable group**	**P value**
No.of eyes/patients	28	25	
Postoperative BCVA at 6 months			
LogMAR (mean ± SD; range)	1.3 ± 0.4 (0.5–2.0)	1.1 ± 0.4 (0.4–2.2)	0.102
Compared with preoperative (*P* value)	*P* = 0.000	*P* = 0.000	
BCVA improvement (mean ± SD)	−1.0 ± 0.7	−1.1 ± 0.7	0.554
MH closure after initial surgery, no. (%)	15 (53.6)	18 (72.0)	0.167
Retinal reattachment after initial surgery, no. (%)	28 (100.0)	24 (96.0)	0.472
Postoperative high IOP, no. (%)	15 (53.6)	11 (44.0)	0.487

*BCVA, best corrected visual acuity; IOP, intraocular pressure; LogMAR, logarithm of the minimum angle of resolution; MH, macular hole*.

**Figure 1:**
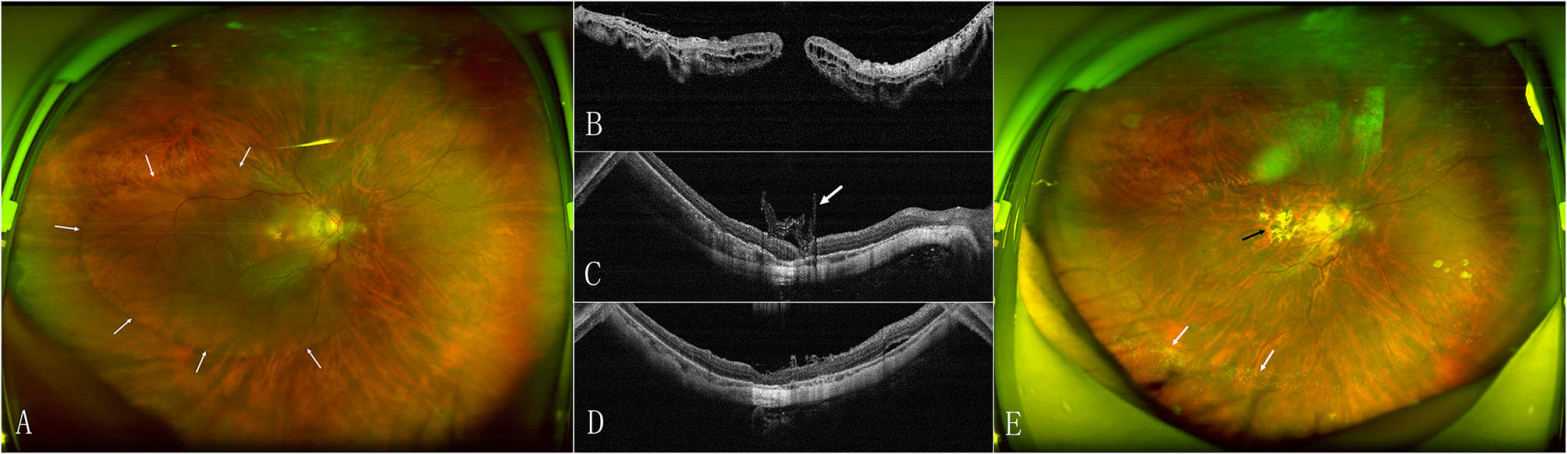
Preoperative and postoperative scanning laser opthalmoscope (SLO) and optical coherence tomography (OCT) images of the eye of a 56-year-old woman with macular hole retinal detachment (MHRD) in the adjustable positioning group. **(A)** SLO shows the retinal detachment (RD) beyond vascular arcade (arrow). **(B)** Preoperative OCT confirms the macular hole (MH). **(C)** OCT shows MH closure and retinal reattachment with inserted internal limiting membrane (ILM) tissue (arrow) plugged into the hole at 2 weeks after surgery. **(D)** OCT shows the foveal microstructure recovery in eyes with MH closure at 6 months. **(E)** SLO shows silicone oil emulsification (white arrow) and chorioretinal atrophic lesion (black arrow) at 6 months.

**Figure 2:**
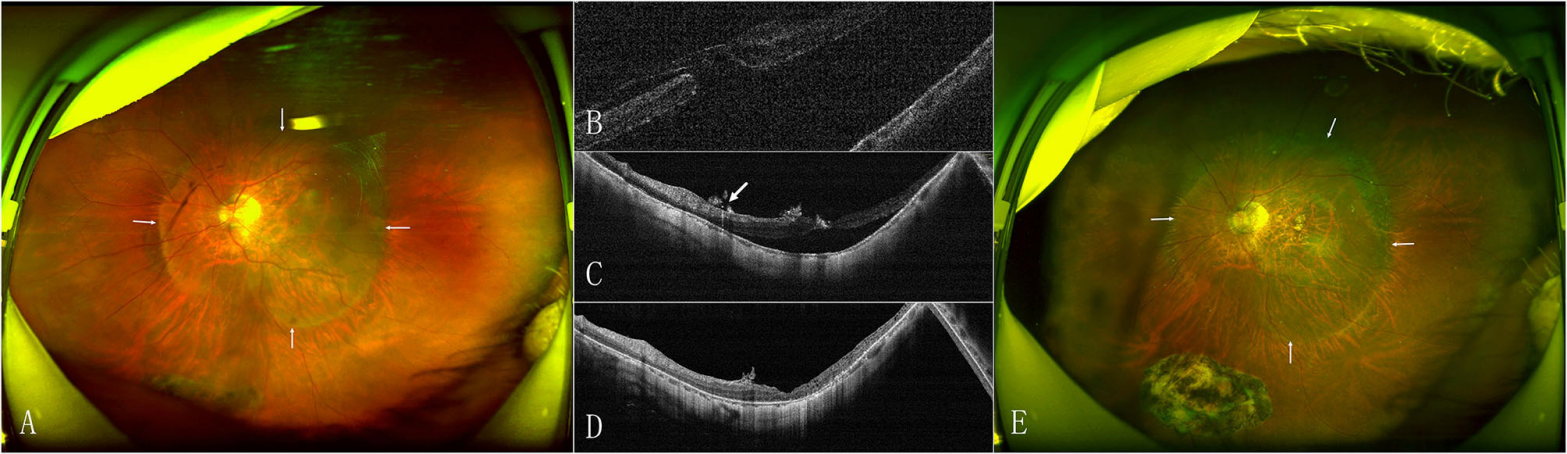
Preoperative and postoperative SLO and OCT images of the eye of a 59-year-old woman with MHRD in the face-down positioning group. **(A)** Preoperative SLO shows that the MHRD beyond vascular arcade to the edge of posterior staphyloma (arrow). **(B)** Preoperative OCT confirms the MH. **(C)** OCT at 12 months after surgery shows that the MH is closed and subretinal fluid in the macular region has not been absorbed (white arrow showing the silicone oil emulsification). **(D)** Twenty seven months later, OCT shows the subretinal fluid is absorbed completely. **(E)** Postoperative SLO at 27 months shows reattached retina and posterior staphyloma (arrow).

**Figure 3:**
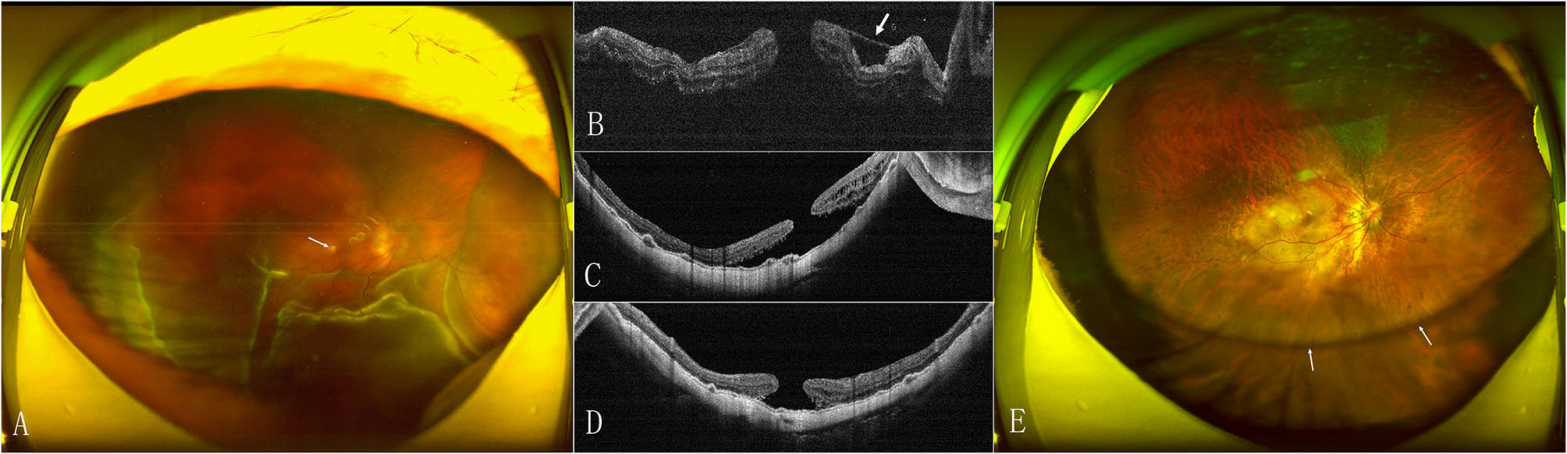
Preoperative and postoperative SLO and OCT images of the eye of a 66-year-old woman with MHRD in the adjustable positioning group. **(A)** Preoperative SLO shows the RD with MH (arrow) beyond vascular arcade. **(B)** OCT confirms the MHRD with an epiretinal membrane (arrow). **(C)** OCT at 6 months after surgery shows that the MH still open and the extent of RD is narrowed. **(D)** Twenty one months later, OCT shows the MH is not closed and the retina around the MH is not reattached. **(E)** Postoperative SLO at 21 months shows that the silicon oil has not been removed yet (arrow).

The MH closed after the initial surgery in 15 (53.6%) eyes in the face-down group and 18 (72%) eyes in the adjustable group (Figure 1). The retinal reattached after initial surgery in 28 (100%) eyes in the face-down group (Figure 2) and 24 (96%) eyes in the adjustable group. There was no significant difference in the rate of MH closure (*p* = 0.167) and retinal reattachment (*p* = 0.472) between the two groups. Only one patient in the adjustable group did not achieve an MH closure and retinal reattachment. During the follow-up, she was unwilling to undergo another surgery, but the extent of retinal detachment gradually narrowed, and the MH was still not closed until 21 months after surgery (Figure 3). There were 15 eyes (53.6%) and 11 eyes (44%) with high IOP after surgery in the two groups (*p* = 0.487). All eyes with high IOP were controlled within the normal range after the treatment with anti-glaucoma drugs, and no surgical intervention was performed. There were no retinal detachment and other complications occurred during the follow-up.

## Discussion

A face-down positioning is a routine requirement for patients after vitrectomy and gas/silicone oil tamponade for RD. But face-down positioning is an important source of discomfort and complications for patients (23, 24), which gradually attracted the attention of the doctor. Chen et al. (32) designed a controlled study to address the issue of positioning after PPV and gas tamponade surgery for rhegmatogenous retinal detachment (RRD). There was no significant difference in the anatomical success rates, BCVA, and the rates of complications between the face-down group and the adjustable positioning group. Martínez-Castillo et al. (31, 33) reported that PPV alone with complete drainage of sub-retinal fluid achieves a high reattachment rate in the management of primary pseudophakic RRD due to inferior retinal breaks. Their patients did not perform a prone position or any other type of positioning during the postoperative period. In the study of Casswell et al. (34), findings suggest that face-down positioning was associated with a reduction in the rate and amplitude of postoperative retinal displacement after macular-involving RD repair and with a reduction in binocular diplopia. Despite this, no association was found with visual acuity or postoperative distortion. However, none of these studies accepted RRD caused by MH in eyes with high degree myopia (−6 diopter or above).

Pars plana vitrectomy (PPV) with ILM peeling and gas tamponade is an important surgical method for the MH. It has been reported that face-down positioning, following an MH surgery, provides no functional or anatomical benefit (36). Some scholars think that a postoperative non-supine positioning is adequate for all the patients with MH after surgery (25–27). Therefore, randomized controlled trials have been conducted to evaluate whether face-down positioning is necessary for recovery from MH surgery (37, 38). The meta-analysis provides sufficient evidence that a non-face-down postoperative positioning is not inferior to a face-down positioning when the MH is smaller than 400 μm. Although a face-down postoperative positioning is highly recommended in MHs larger than 400 μm, the ideal visual improvement rate was not influenced by postoperative positioning (37). Zhu et al. (39) used a novel surgical protocol using vitrectomy, ILM peeling, and autologous blood clot covering the MH at the end of the MH surgery, which eliminated the gas tamponade and thus, the need for postoperative face-down positioning. Complete MH closure was achieved in all 18 eyes at the end of the follow-up period, and among them were five large MHs (minimum diameter > 400 mm).

Among myopic patients with MH, the incidence of RD increased as myopia worsened (1.1% RD in myopia under −3 D, 67.7% RD in myopia between −8, and −3.25 D, and 97.6% RD in myopia over −8.25 D) (1). AL elongation and posterior staphyloma contribute to the disparity in the length of the retina and the RPE-choroid-sclera complex, leading to the progression of retinal detachment (1, 2). The articles on MHRD surgery published in the past 5 years are mostly from East Asia (7–20) (as shown in Table 3). A total of 404 eyes in 403 patients were observed, including 331 women (82.1%) and 72 men (17.9%), and the majority of female patients were similar to the population data (40, 41). If vitrectomy and ILM peeling have become the standard treatment for MHRD with a retinal reattachment rate of 91.5%, then the inverted ILM flap groups can achieve 97.3% (42). Otherwise, the inverted ILM insertion technique seems to improve the anatomical results in terms of MH closure rate with there being a tendency for better postoperative visual acuity in the inverted ILM insertion group (43). In these studies, none of them gave up the face-down positioning, and after filling with gas or silicone oil, the patients were asked to keep a prone positioning for 1–2 weeks and avoid supine positioning afterward.

**Table 3:** Published studies about macular hole retinal detachment (MHRD) in the last 5 years.

**References**	**Country/Region**	**Groups**	**No. of eyes**	**Female/Male**	**Age**	**AL**	**RD within/ beyond the arcade**	**PPV procedure**	**ILM dying**	**Tamponade agent**	**Postoperative positioning**	**Initial MH closure**	**Initial retinal reattachment**
Gu et al. (19)	China	Inverted ILM flap	22	20/2	61.5 ± 8.6	28.28 ± 2.01	/	23G/25G	ICG	C3F8/SO	Maintain a facedown position for 1 week.	20 (90.9%)	22 (100%)
		ILM peeling	20	19/1	63.6 ± 8.2	28.34 ± 1.78						8 (40%)	19 (95%)
Zhu et al. (20)	China	Inverted ILM insertion	26	21/5	63 ± 5	27.82 ± 2.20	6/20	23G	ICG	Air/SO	A prone position for ≥ 8 h per day for 1 week	19 (73%)	26 (100%)
		Inverted ILM flap	23	16/7	60 ± 8	28.92 ± 2.86	6/17	23G	ICG	Air/SO		20 (87%)	21 (91%)
Kim et al. (18)	Korea	Inverted ILM flap	3	21/1	61.8 ± 10.1	28.96 ± 1.57	/	20G/23G /25G	ICG/BBG	SF6/C3F8/SO	Stay in a strict face-down position for 1 to 7 days after surgery	2 (66.7%)	3 (100%)
		ILM peeling	19									11 (57.9%)	19 (100%)
Ho et al. (15)	Taiwan, China	Inverted ILM flap	18	14/3	60.2 ± 8.2	29.25 ± 2.10	4/14	23G/25G	ICG	C3F8	Keep a prone position for 1 week and avoid a supine position afterwards	18 (100%)	17 (94%)
Chen et al. (14)	Taiwan, China	Inverted ILM flap	13	8/5	65.5 ± 7.7	29.75 ± 2.21	10/3	23G/25G	ICG	SF6/C3F8	Keep in a facedown position over night and no supine for 1 week	13 (100%)	13 (100%)
		ILM peeling	14	9/5	62.4 ± 8.6	29.45 ± 1.58	12/2	23G/25G	ICG	SF6/C3F8		6 (42.9%)	14 (100%)
Wakabayashi et al. (17)	Japan	Inverted ILM insertion	13	11/2	67.8 ± 9.9	29.4 ± 0.9	5/8	20G/23G /25G	TA/ICG /BBG	SF6/C3F8/S	/	12 (92%)	12 (92%)
		ILM peeling	36	34/2	69.2 ± 9.1	29.6 ± 1.7	7/29	20G/23G /25G	TA/ICG /BBG	SF6/C3F8/SO		14 (39%)	31 (86%)
Takahashi et al. (16)	Japan	Inverted ILM flap	16	14/2	68.4 ± 7.8	29.1 ± 1.9	8/8	23G/25G /27G	BBG	SF6/C3F8/SO	Maintain a facedown position for mean 12 days	12 (75%)	13 (81%)
		ILM peeling	16	15/1	69.1 ± 8.5	29.6 ± 1.1	9/7	23G/25G /27G	BBG	SF6/C3F8/SO		4 (25%)	15 (93%)
Xu et al. (13)	China	Inverted ILM flap	18	15/3	60.17 ± 9.04	27.37 ± 0.91	10/8	23G	BBG	C3F8	Maintain a facedown position for 3 to 4 weeks	16 (89%)	18 (100%)
		ILM peeling	17	13/4	59.47 ± 8.53	27.71 ± 0.81	12/5	23G	BBG	C3F8		8 (47%)	17 (100%)
Kinoshita et al. (11)	Japan	Inverted ILM flap	5	3/2	64.4 ± 4.5	31.76 ± 2.38	/	25G	BBG	SF6/C3F8	Maintain a facedown position for 10 to 14 days.	5 (100%)	5 (100%).
Sasaki et al. (12)	Japan	Inverted ILM flap	6	5/1	75.0 ± 6.4	30.47 ± 2.57	1/5	25G	BBG	SF6/C3F8	Maintain a facedown position postoperatively for at least 5 days	6 (100%)	6 (100%)
		ILM peeling	9	7/2	66.0 ± 12.5	30.10 ± 1.95	3/6	25G	BBG	SF6/C3F8		5 (55.5%)	5 (55.5%)
Baba et al. (10)	Japan	Inverted ILM flap	10	5/5	74	28.95	6/4	25G	BBG	C3F8	Maintain a prone position for approxi mately 5 days after surgery	8 (80%)	10 (100%)
		ILM peeling	11	8/3	68	30.30	7/4	25G	BBG	C3F8		4 (36%)	10 (91%)
Chen et al. (8)	Taiwan, China	Inverted ILM insertion	20	16/4	62.06 ± 8.90	28.40 ± 1.94	11/9	23G	ICG	C3F8	Keep in a facedown position over night and no supine for 1 week	20 (100%)	20 (100%)
		ILM peeling	20	14/6	60.53 ± 8.78	29.35 ± 1.88	10/10	23G	ICG	C3F8		7 (35%)	20 (100%)
Matsumura et al. (9)	Japan	Inverted ILM flap	10	8/2	67.7 ± 9.7	28.4 ± 2.2		25G	BBG	SF6/C3F8/SO	Maintain a prone position postoperatively for at least 1 week	9 (90%)	9 (90%)
		ILM peeling	12	11/1	75.3 ± 8.7	30.4 ± 1.6		25G	BBG	SF6/C3F8/SO		4 (33.3%)	6 (50.0%)
Lai et al. (7)	Taiwan, China	Inverted ILM insertion	27	24/3	59.1 ± 10.6	29.37 ± 1.92	9/18	23G	ICG	C3F8	Remain in a prone position for 1 day and to avoid the supine position afterward	26 (96%)	26 (96%)

*AL, axial length; BBG, brilliant blue G; C3F8, perfluoropropane; ICG, indocyanine green; ILM, internal limiting membranes; MH, macular hole; PPV, pars plana vitrectomy; RD, retinal detachment; SF6, sulfur hexafluoride; SO, silicon oil; TA, triamcinolone acetonide*.

Previous studies have reported that the healing of MHs begins within 24 h after the surgery, and the bridge configuration occurs around 3 days thereafter (44). The MHs were basically healed within 3 days after surgery, and those that were not healed within 3 days were still open during the 3-month follow-up (45). Seno et al. (46) observed and scored the compliance of the face-down positioning four times per day for 3 days post-surgery for patients who had undergone a primary vitrectomy and gas tamponade for MH or RRD. In fact, the compliance with the face-down positioning was considerably varied among patients, and some patients failed nearly or more than half the time, with considerable variation among patients and better adherence by the female patients, but without associations to the outcomes. When the eye is filled with gas or silicone oil after surgery, the tamponade agent can keep contact with the retina, exert the effect of surface tensions, and close the hole or break except in the lowest position of the vitreous cavity. The macular hole is located at the posterior pole of the eyeball and is not at the lowest position in the eye except during supine positioning. Silicone oil can close the macular hole in the non-facedown positioning. Based on these, it is enough for patients to avoid supine positioning.

To the best of our knowledge, this is the first study to address the issue of positioning after PPV and silicone oil tamponade surgery for MHRD. There was no significant difference in the MH closure, retinal reattachment, and postoperative BCVA between the face-down group and adjustable group in our study. In addition, better postoperative BCVA was gained in both the groups. The rate of MH closure and retinal reattachment was similar to previous reports (42). No complications occurred after the operation.

There are several limitations in our study that should be addressed. First, the study was non-randomized and lacked a randomized model for the positioning choice. Second, the follow-up period was probably short to observe the full recovery of foveal micro-structures. Third, all the eyes were tamponade by silicon oil because we lack the supply of C3F8.

In conclusion, the results of this study suggest that adjustable positioning after PPV with silicon oil tamponade for MHRD repair is effective and safe, and choosing an adjustable positioning over a face-down positioning approach does not reduce the possibility of MH closure, retinal reattachment, and improvement of visual acuity or significantly increase the risk of complications. Face-down positioning does not seem to be necessary for all patients with MHRD. A larger and prospective randomized controlled trial study is recommended to determine the long-term outcomes of adjustable positioning after PPV surgery for MHRD.

## Data Availability Statement

The raw data supporting the conclusions of this article will be made available by the authors, without undue reservation.

## Ethics Statement

The studies involving human participants were reviewed and approved by Institutional Review Board of Qingdao Eye Hospital of Shandong First Medical University. The patients/participants provided their written informed consent to participate in this study.

## Author Contributions

Material preparation, data collection, and analysis were performed by YG and TR. The first draft of the manuscript was written by JL. All authors contributed to the conception and design of the study, provided comments on the manuscript, and read and approved the final manuscript.

## Funding

The funding was provided by the Qingdao Science and Technology Demonstration and Guidance Project (20-3-4-45-nsh) and the Academic Promotion Plan of Shandong First Medical University & Shandong Academy of Medical Sciences (2019ZL001).

## Conflict of Interest

The authors declare that the research was conducted in the absence of any commercial or financial relationships that could be construed as a potential conflict of interest.

## Publisher's Note

All claims expressed in this article are solely those of the authors and do not necessarily represent those of their affiliated organizations, or those of the publisher, the editors and the reviewers. Any product that may be evaluated in this article, or claim that may be made by its manufacturer, is not guaranteed or endorsed by the publisher.
